# Effects of paddles and fins on front crawl kinematics, arm stroke efficiency, coordination, and estimated energy cost

**DOI:** 10.3389/fphys.2023.1174090

**Published:** 2023-05-22

**Authors:** C. C. De Matos, B. Guignard, F. De Souza Castro, A. Guimard

**Affiliations:** ^1^ Aquatic Sports Research Group, Universidade Federal do Rio Grande do Sul, Porto Alegre, Brazil; ^2^ Université Rouen Normandie, CETAPS UR 3832, Rouen, France; ^3^ Université Sorbonne Paris Nord, Hypoxie et Poumon, H&P, INSERM, UMR 1272, Bobigny, France; ^4^ Département STAPS, Université Sorbonne Paris Nord, Bobigny, France

**Keywords:** swimming equipment, underwater kick, stroke cycle, propulsive time, propulsive phases, non-propulsive phases, freestyle swimming technique, energy cost

## Abstract

Paddles and fins are used in swimmers training with different objectives (e.g., increase propulsive areas of hands and feet, improve the feeling of water flow). These artificial modifications of the stroke might be viewed as external constraints of the stroke task, both will either disturb or facilitate swimming modalities, so the coaches should manipulate its use to extract benefits for performance. This study seeks to investigate the precise effects of wearing either paddles (PAD) or fins (FINS) vs. a no-equipment (NE) trial in three all-out front crawl exercises on swimmer kinematics, arm stroke efficiency ( ηp ), upper-limbs coordination patterns (Index of Coordination, IdC), and estimated energy cost (C). Eleven regional to national-level male swimmers participated in the study (age: 25.8 ± 5.5 years, body mass: 75.2 ± 5.5 kg, height: 177 ± 6.5 cm) and were recorded from both sides of the swimming pool to collect all variables. Repeated measures ANOVA and Bonferroni *post hoc* were used to compare the variables. Effects sizes were calculated. Time to cover the distance and velocity were higher in FINS swimming, with larger values of stroke length (SL) and lower kick amplitude in comparison to the other trials (PAD and NE). The use of FINS also modified the stroke phases durations by presenting significant lower propulsion time during the stroke in comparison to PAD or NE. Values of IdC were lower (IdC < −1%, so catch-up pattern of coordination) for FINS in comparison to NE. In terms of ηp , using PAD or FINS demonstrate higher arm stroke efficiency than swimming without equipment. Finally, C was significantly higher in FINS swimming in comparison to NE and PAD. From the present results, it should be noted that the use of equipment such as fins deeply modify the structure of the swimming stroke (from the performance-related parameters through the kinematics of both upper and lower limbs to the stroke efficiency and coordination pattern). So, using equipment should be appropriately scaled by the coaches to the objectives of the training session in swimming, and in emergent sports such as “SwimRun”, paddles and fins must be viewed as tools to achieve higher velocities to cover a given distance.

## 1 Introduction

Swimming equipment is used during training for technique, in a physiological way, but also to enlarge the swimming conditions encountered (e.g., variations of sensations to reduce monotony of the session). Using paddles or fins can lead to spatiotemporal changes in the swimming cycle that can have an influence both on the production of propulsive forces and on swimming technique. Since paddles and fins need less physiological demands (blood lactate concentration and rate of perceived exertion) for the same swimming speed without equipment (Matos et al., 2013), the best gains related to the use of both should occur when swimming training is performed at higher intensities, for a longer period. When swimming speed is increased (to supramaximal speeds) with the equipment, drag increases in a square function of the speed increased, so that to overcome this drag, the swimmer must apply more force in the water. Thus, the metabolic demand is greater in response to paddles/fins use (in speed next to maximal or supramaximal). Through this stimulus, the athlete tends to promote a reorganization in the swimming pattern, such as increase of both propulsive phase durations and stroke length, and decrease of stroke rate, due to changes in the development of both strength and coordination patterns (Schnitzler et al., 2011). Schnitzler et al. (2011) used a parachute attached to the swimmer in a flume: they evidenced that at higher speeds, the stroke rate (SR) and index of coordination (IdC) raised, explained by a significant decrease of the glide in benefits to the pull duration. Therefore, we can see that the changes in swimming speed with equipment compared to swimming without equipment come from changes in the reorganization of the percentage duration of the stroke phases. For instance, Gourgoulis et al. (2009) showed that large paddles led to a significant increase of the entry and catch phase relative duration (35.5% ± 4.9%) in comparison to small paddles (33.6% ± 3.5%) and no paddle (33.7% ± 4.7%). This results in a significant decrease in propulsive phases relative durations. In this way, the use of equipment should be finely supervised so that the greatest possible adaptation occurs and, consequently, the benefits of training with paddles, parachutes, fins, or others may be transferred in swimming competitions.

The upper limbs provide approximately 90% of the propulsion in front crawl swimming (Deschodt et al., 1999). Paddles are often used in swimming training sessions with different goals, such as increasing strength and endurance conditional capabilities. Still, we can also mention the increase in the surface area of the hand and, consequently, the increase in the contact surface with the water to propel the body forward (Gourgoulis et al., 2006; Gourgoulis et al., 2008; Gourgoulis et al., 2009). Thus, for the same stroke rate, in males, when swimming without and with paddles (paddles’ area: 360 cm^2^) were compared, it was observed that the equipment decreased the percentage duration of the non-propulsive phases of the stroke, modifying the pattern of coordination of the swimming stroke, changing from catch-up (i.e., lag time between two consecutive actions of the arms) to opposition (continuity in the propulsive actions, Chollet et al. (2000).

Despite their lower contribution in the generation of propulsion (around 10%, according to Deschodt et al. (1999), the lower limbs actions affect the swimmer’s energy cost. It tends to assist in improving horizontal alignment of the body, consequently decreasing the active drag during swimming (Yanai, 2001). As a result, wearing fins would increase this leg raising at velocities superior to 1.6 m/s. But, in the meantime, the continuous kicking action visible during sprints may raise the energy cost of swimming, as stated by Zamparo et al. (2020). In turn, it will tend to affect arm stroke efficiency (as an increase in the ratio of total work produced by the swimmer to useful work, that which actually takes the swimmer forward; di Prampero, 1986) by increasing swimming speed and decreasing stroke rate (Matos et al., 2013). To go deeply, the fin design (size, but also flexible vs. rigid materials that composed the fins) may also impact kinematics and propulsive swimming efficiency (Zamparo et al., 2002).

Therefore, the aims and originality of the present study are to measure the precise role played by the use of paddles and fins on the efficiency and motor control (coordination pattern) of the stroke (macroscopic view), on the biomechanics (limbs kinematics and stroke phases, leg kicking pattern; microscopic view), and on the integrated parameter (between physiology and biomechanics)—the estimated energy cost over a 50 m all-out front crawl test. All sport classes are confronted with this event, in which a compromise should occur between the technique and high speeds (involving increased drag). Thus, we investigated in what manner an upper limb equipment (i.e., paddles), or a lower limb equipment (i.e., fins) may challenge the complete front crawl swim at maximal intensity for the same swimmers. We hypothesized that such a swimming intensity would reveal a large panel of motor adaptations/alterations that may be transferred during competitions without equipment. This would offer insights on the precise roles of such equipment to encourage its use at specific moments of training season, with well-defined functions.

## 2 Materials and methods

### 2.1 Participants

Eleven well-trained male swimmers, from regional to national level, 61.6% ± 10.2% from the current world record of the 50-m freestyle; 528 ± 103.9 FINA points, participated in the study (age: 25.8 ± 5.5 years, body mass: 75.2 ± 5.5 kg, height: 177 ± 6.5 cm and arm span: 185 ± 7.2 cm). Swimmers had at least 7 years of training (12.4 ± 7.4 years) and at least 2 years using paddles and fins in training sessions (4.6 ± 2.0 years). The participants had to be at least 18 years old. Four of them declared to be sprinters (50- and 100-m freestyle), two middle distance swimmers (200- and 400-m freestyle), and five were long-distance swimmers (800- and 1500-m freestyle). Swimmers who had not previously trained for more than 4 weeks or who developed injuries or illnesses during the study were excluded. This sample includes different swimmers’ profiles to be representative of a classic training group. All of them were informed and familiarized with the methodological procedures and signed an informed consent form. The research was approved by the Research Ethics Committee of the Universidade Federal do Rio Grande do Sul (number 20442).

### 2.2 Protocol

Swimmers were tested along three non-consecutive days, with a 24 h rest period in between. On the first day, anthropometric features were measured (body mass, height, and arm span). Body mass and height measurements were then used to estimate the areas of the hand palm and foot (149 ± 10.9 cm^2^; 388 ± 28.6 cm^2^ respectively). To estimate the surface area of the hand, we used the equation proposed by Du Bois and Du Bois (1916) multiplied by 0.78% [mean value of the surface area of the hand proposed by Amirsheybani et al. (2001)]:

$$Hand\;surface\;area=\left(0.007184*{body\;mass}^{0.425}*{height}^{0.725}\right)*0.78\%$$

To estimate the surface area of the foot we used the equation proposed by Du Bois and Du Bois (1916) multiplied by 2.03% [mean value of the surface area of the foot proposed by Yu and Tu (2009)]:

$$Foot\;area=\left(0.007184*{body\;mass}^{0.425}*{height}^{0.725}\right)*2.03\%$$

**FIGURE 1:**
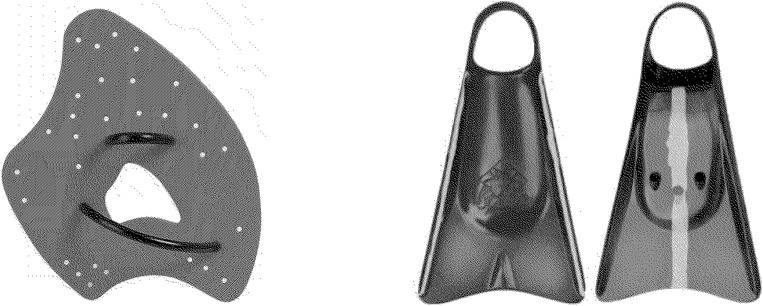
Catalyst TYR^®^ paddle (Huntington Beach, CA, United States), Kpaloa^®^ fins (Rio de Janeiro, RJ, BR).

On the same day, the participants performed a front crawl 50-m test with no-equipment (NE) as a baseline. During the following 2 days, two randomized tests were performed with paddles (PAD) or fins (FINS) conditions. The used equipment was: (i) a Catalyst TYR^®^ (Huntington Beach, CA, United States) in high-density polyethylene with an area of 300 cm^2^ for paddles, and (ii) a Kpaloa^®^ (Rio de Janeiro, RJ, BR) in vulcanized rubber with flexible flaps on the sides, small and semi-rigid model of 488 cm^2^ of area for fins. The equipment are presented in Figure 1.

**FIGURE 2:**
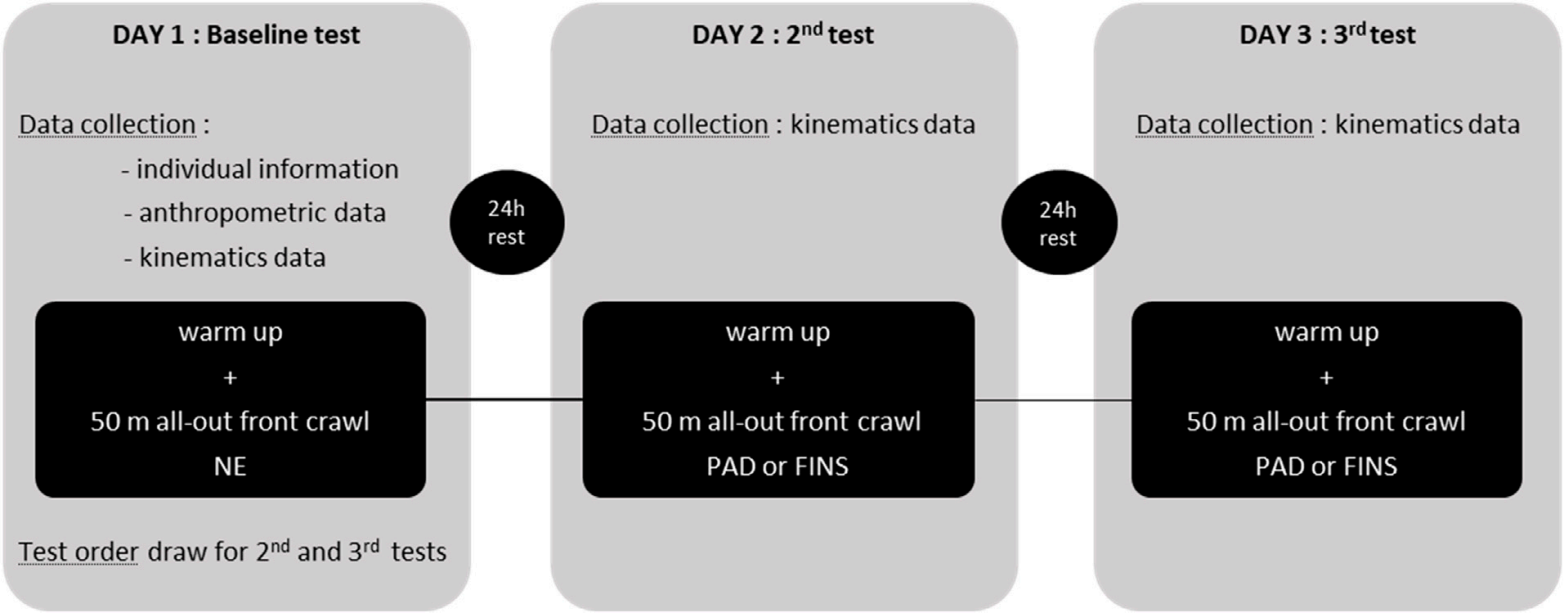
Study design (NE = no-equipment; PAD = paddles condition; FINS = fins condition).

The warm-up was standardized in 800-m front crawl at low intensity with the last 200 m performed with the respective equipment on paddles and fins days. After the warm-up, the 50-m front crawl test was performed at maximum velocity, with an in-water start and underwater glides on each swimming lap limited to 5 m, in a heated 25-m pool (average water temperature: 29°C ± 0.5°C), 2-m deep. The tests were always programmed between 2:00 and 4:00 PM. Athletes were asked not to train on the assessment days and to abstain from intense physical effort and/or training for 48 h before the tests. Regarding the nutritional aspects, all the swimmers were asked to keep the same nutritional intake all over the experiment. Figure 2 shows the study design.

### 2.3 Data collection and analysis

**FIGURE 3:**
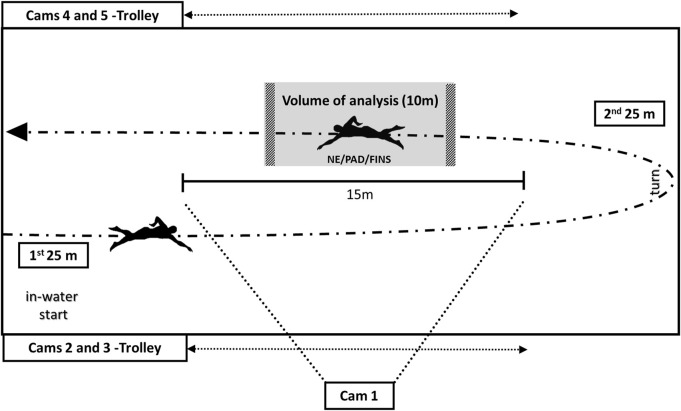
Set-up for data collection. The volume of analysis of 10 m is highlighted in grey with initial and final cross-hatched marks.

The swimmers’ images throughout the tests were obtained with five time-synchronized video cameras operating at 60 Hz (Sanyo VPCWH1 XACTI TH1, Japan). Therefore, the maximal resolution of those cameras was 0.0167 s for the computation of each temporal variable described below. The first camera was fixed at the side edge of the pool (half the length of the pool), 3 m high, and 15.5 m (linear distance) from the swimmer as he passed through the center of the pool. On each side of the pool two cameras were placed on manually moved trolleys along moving tracks of 15 m (cameras 2 and 3 in one pool side, and cameras 4 and 5 in the opposite pool side), one camera 30 cm below the surface and the other 10 cm above the surface. Each pair was 7.5 m away from the swimmer and the optical axis was parallel to the transverse axis passing through the femoral head (sagittal view). These distances, for the fixed camera, allowed a 15 m field of view of the swimmer’s displacement plane for a final volume of analysis corresponding to 10 m (Figure 3).

Only the second 25 m was used as representative of the test to identify the effect of the equipment more accurately on the analyzed variable, reducing the number of independent variables. In this section, the central demarcation of the whole pool ensured pure swimming analysis (Ribeiro et al., 2010) (i.e., 10 m in the present study) and avoided biases from the wall pushes after the turns. For the five cameras, two calibrators were used. The first was 1 m long, positioned vertically from the water surface, at the center of the 10 m (at 12.5 m of the pool). The second was 1.5 m long, positioned horizontally, at the water surface, in the swimmer’s displacement plane, at 12.5 m of the course. The calibrators were positioned in the lane where the tests were carried out and recorded (3 s) before each test. The number of pixels that comprised the length of the calibrators for all cameras, underwater and at the surface, was identified. A video annotation tool software was used (Kinovea v0.8.15 open-source software—www.kinovea.org) to analyze the kinematics variables.

Time to cover the imposed distance was controlled during the all-out test (as the time in each 50 m test with an in-water start) by an experienced timekeeper using a manual stopwatch (Casio HS-30W, Japan). To obtain the mean swim velocity (v), the initial and final 7.5 m of the second 25 m were removed. In this way, it is possible to analyze only the 10 m of swimming without changes in the swimming mechanics due to start and turn. Markings were made along the 25 m of the pool to analyze only the central 10 m. The v was quantified by the quotient between 10 m and the time to cover the 10 m (the swimmer’s head passing through the initial and final markers of the 10 m). Stroke rate (SR) was quantified by the quotient between 3 stroke cycles and the time to perform them, within the demarcated 10 m. The average distance covered by the body in each cycle (stroke length—SL) was calculated by the quotient between v and SR.

Kick rate (KR), depth (KD) and vertical amplitude (AMP) were obtained by analyzing the kicking underwater images. KR was identified by the number of kick cycles (a descending and an ascending movement performed by one of the lower limbs, see Zamparo et al., 2002) throughout a stroke cycle. KD was considered as the greatest distance between the tibia lateral malleolus and the waterline over a complete stroke cycle. AMP was quantified by the greatest vertical distance between the lateral malleolus of the lower limbs throughout a kick cycle, discarding the kicking cycle with rotational movements for breathing as previously proposed (Zamparo et al., 2002; 2006).

The duration of the stroke phases (water entry and catch, pull, push, and recovery—relatives to the duration of a stroke cycle - %) and the index of coordination (IdC) were obtained through the identification of four key points of the stroke (water entry, catch when hand starts its backward movement, shoulder plane during the transition from pull to push and water exit) (Chollet et al., 2000). From these key moments, the four phases of the stroke were identified and the lag time between two consecutive propulsive actions of each upper arm was quantified. As the main aim of this study was to verify the paddles and fins effects, the mean values from the right and left single strokes were used in the analysis. The images were qualitatively and independently assessed by three previously trained researchers. These evaluators used the same criteria described by Chollet et al. (2000). Through the analysis of the IdC, according to Chollet et al. (2000) and Seifert (2010), three coordination patterns were defined: opposition (IdC between −1% and 1%); catch-up (IdC < −1%) and superposition (IdC >1%). All stroke phase durations and IdC were identified on both sides of the body. The time of the propulsive phases was obtained by the sum of the pull and push phases, whereas the duration of the non-propulsive phases was the sum of the water entry and catch and recovery phases. The duration of the complete stroke was therefore the sum of the propulsive and non-propulsive phases (from one hand entry to the next entry of the same hand). The IdC1 was defined as the time between the end of the propulsive phase of the stroke on the left body side and the beginning of the propulsive phase of the right body side, reported to the cycle duration. The IdC2 was defined as the time between the end of the propulsive phase of the right body side and the beginning of the propulsive phase of the left body side, reported to the cycle duration. The mean of IdC1 and IdC2 was calculated as the IdC.

The total propulsion time (Tprop) over the 10 m volume of analysis was identified as previously proposed (Alberty et al., 2009), according to Eq. 1:

$$Tprop=Tcycle\left(100\%+2IdC\right)*10/SL$$

Where Tcycle is the mean cycle duration, IdC is the mean index of coordination, 10 the distance (in m), and SL is the mean stroke length (m). The arm stroke efficiency $\left(\eta p\right)$ , in %, was calculated by the simplified model previously proposed by Zamparo et al. (2005), described according to Eq. 2:

$$\eta p=\left(\left(\frac{v*0.9}{2*\pi *SR*L}\right)*\frac{2}{\pi }\right)*100$$

Where ηp is the arm stroke propelling efficiency (in %), v is the mean swimming velocity, SR the mean stroke rate and L the linear distance between the center of the shoulder and the center of the hand when the hand is exactly below the shoulder, in the pull-push phases transition, and assumed as 0.52 m (Zamparo et al., 2005; Castro et al., 2021). The energy cost (C) was estimated individually from the *v*, for each condition (NE, PAD, FINS) by applying the exponential regression equation (Eq. 3) previously reported (Caputo et al., 2006):

$$C=0.4882+0.0561e^{\left(\frac{v}{0.5981}\right)}$$

Where C is the estimated energy cost (in kJ/m), and *v* is the mean swimming velocity.

### 2.4 Statistical analyses

The required sample size was estimated *a priori* with the following assumptive parameters, using G*Power version 3.1.9.7 (Faul et al., 2009) (Düsseldorf University, Düsseldorf, Germany): (a) F test for one group and three measurements (each swimming condition); (b) effect size of 0.45 (Barbosa et al., 2013); (c) alpha-value of 0.05; (d) statistical power of 0.80; and (e) correlations between measures of 0.5. The calculated estimated required sample size was 10, which is lower than the actual sample size (*n* = 11). Data normality was not tested since ANOVA is robust to normality infractions (Sawyer, 2009). Descriptive statistics (mean ± standard deviation) and lower and upper limits (95%) of the confidence intervals of the mean were calculated. Repeated measures ANOVA, with Bonferroni pairwise comparisons, was used to compare the variables among the conditions. Mauchly’s test was used to analyze sphericity, and when it was not assumed, Epsilon Greenhouse-Geisser correction was applied (degrees of freedom corrected). The independent variables analyzed were trials with (i) NE, (ii) PAD, and (iii) FINS. The dependent variables analyzed were time to cover the imposed distance, v, SL, SR, KR, KD, AMP, stroke phases duration, ηp , Tprop, and IdC, and C. The partial eta^2^ was used as an indicator of the effect size, following the criteria: 0.02–0.13 small; 0.14–0.26: medium; >0.26: large (Cohen, 1988). The SPSS 22.0 statistical package was used in all statistical analyses, for alpha <0.05.

## 3 Results

There was a significative and strong (according to the ES criteria) effect of equipment on the time to cover the imposed distance (F_2,20_ = 77.2; *p* < 0.001; partial eta^2^ = 0.89). Time to cover the imposed distance was lower in FINS condition (25.7 ± 1.3 s) when compared to PAD (28.2 ± 1.9 s; *p* < 0.001) and NE (8.5 ± 2.1 s; *p* < 0.001) with no significant difference between the latter two conditions. There was a significative and strong (according to the ES criteria) effect of equipment on v, over the central 10 m, (F_1.3, 13.1_ = 15.6; *p* < 0.001; partial eta^2^ = 0.61). The v was higher in FINS (2.10 ± 0.18 m/s) than both PAD (1.86 ± 0.15 m/s; *p* = 0.003) and NE (1.71 ± 0.24 m/s; *p* = 0.005), with no significant difference between the latter two conditions. Arm SL and SR, KR, KD, and kick AMP results are presented in Table 1. In FINS, arm SL and kick AMP were significantly higher and lower, respectively, than NE and PAD, and KD was significantly lower than PAD. In PAD, arm SL and SR were significantly higher and lower, respectively, than NE.

**TABLE 1:** Mean ± SD and lower and upper limits (95%) of the confidence intervals of Arm SL, Arm SR, KR, KD, and AMP in the no-equipment (NE), paddles (PAD) and fins (FINS) conditions. Statistical results F (degrees of freedom), p, and partial eta^2^;^a,b^: significant difference with NE, PAD condition respectively.

	NE	PAD	FINS	*F*_(2, 20)_ *p*	Partial eta^2^
**Arm SL (m)**	2.02 ± 0.20 [1.88 to 2.16]	2.40 ± 0.33^a^ [2.17 to 2.62]	2.46 ± 0.50^a^ [2.12 to 2.79]	8.4 0.002	0.46
**Arm SR (cycles∙min** ^**-1**^ **)**	58 ± 4.9 [54 to 61]	51 ± 7.0^a^ [46 to 55]	55 ± 8 [49 to 60]	6.9 0.005	0.41
**KR (Hz)**	2.44 ± 0.40 [2.17 to 2.70]	2.23 ± 0.58 [1.84 to 2.61]	2.35 ± 0.41 [2.07 to 2.61]	1.04 0.37	0.09
**KD (m)**	0.36 ± 0.06 [0.32 to 0.39]	0.38 ± 0.06 [0.33 to 0.41]	0.32 ± 0.08^b^ [0.26 to 0.37]	3.64 0.045	0.27
**AMP (m)**	0.34 ± 0.03 [0.32 to 0.36]	0.35 ± 0.03 [0.33 to 0.36]	0.29 ± 0.02^a^ [0.27 to 0.30]	19.5 < 0.001	0.66

SL, stroke length; SR, stroke rate; KR, kick rate; KD, kick depth; AMP, kick vertical amplitude.

At the scale of one stroke cycle, the percentage durations of each stroke phase are presented in Table 2. In FINS, pull and recovery durations were significantly lower and higher, respectively, than in NE, and entry and catch duration was higher than in PAD. The FINS condition leads to a significantly shorter propulsive phase duration than NE (*p* < 0.05).

**TABLE 2:** Mean ± SD and lower and upper limits (95%) of the confidence intervals of the stroke phase’s duration in the no-equipment (NE), paddles (PAD), and fins (FINS) conditions. Statistical results F (degrees of freedom), p, and partial eta^2^.^a,b^: significant difference with NE, PAD condition respectively.

	NE	PAD	FINS	*F*_(2, 20)_ *p*	Partial eta^2^
**Entry and catch (%)**	41.1 ± 7.4 [36.0 to 46.0]	41.9 ± 6.5 [37.5 to 46.2]	46.9 ± 3.0^a,b^ [44.8 to 48.9]	4.96 0.018	0.33
**Pull (%)**	13.6 ± 5.2 [10.0 to 17.0]	11.8 ± 5.5 [8.1 to 15.4]	7.3 ± 3.1^a^ [5.2 to 9.3]	5.60 0.012	0.36
**Push (%)**	26.2 ± 3.1 [24.1 to 28.3]	24.7 ± 3.8 [22.1 to 27.2]	24.4 ± 4.8 [21.1 to 27.6]	1.00 0.39	0.09
**Recovery (%)**	19.1 ± 2.6 [17.3 to 20.9]	21.7 ± 2.8 [19.7 to 23.5]	23.3 ± 4.7^a^ [20.1 to 26.4]	6.28 0.008	0.39
**Propulsive (%)**	39.8 ± 5.9 [35.8 to 43.7]	36.4 ± 6.5 [32.4 to 40.8]	31.6 ± 4.8^a^ [28.1 to 34.8]	8.47 0.002	0.46
**Non propulsive (%)**	60.2 ± 5.8 [56.2 to 64.1]	63.6 ± 6.5 [59.2 to 67.9]	68.4 ± 4.8^a^ [65.1 to 71.5]	8.53 0.002	0.46

**FIGURE 4:**
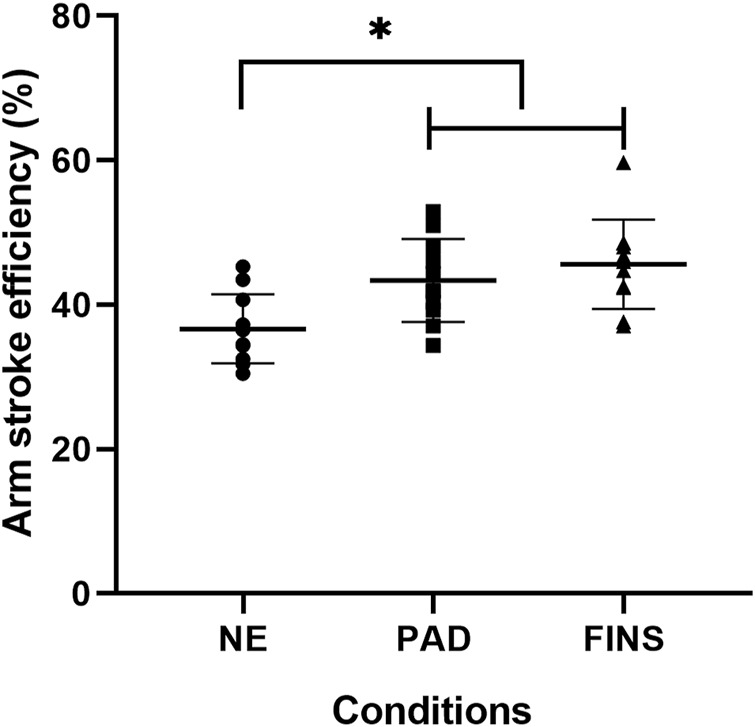
Mean ± SD of arm stroke efficiency ( ηp , in %) in the no-equipment (NE), paddles (PAD), and fins (FINS) conditions; F_(2, 20)_ = 10.24, *p* = 0.001; partial eta^2^ = 0.51. * = NE significantly lower than PAD and FINS at *p* < 0.05.

**FIGURE 5:**
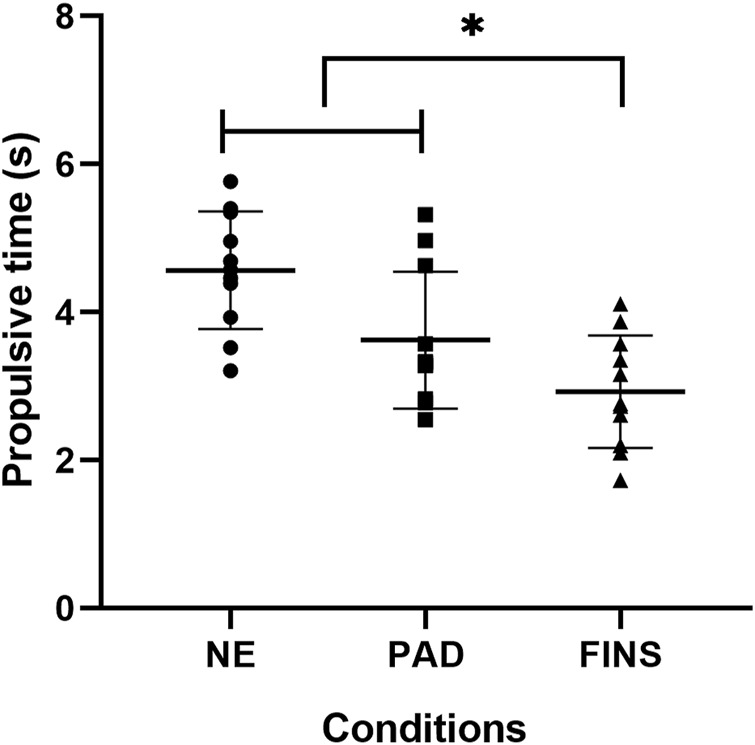
Mean ± SD of total propulsive time (Tprop, in s) in the no-equipment (NE), paddles (PAD), and fins (FINS) conditions; F_(2, 20)_ = 15.0; *p* < 0.001; partial eta^2^ = 0.60. * = FINS significantly lower than NE and PAD at *p* < 0.05.

**FIGURE 6:**
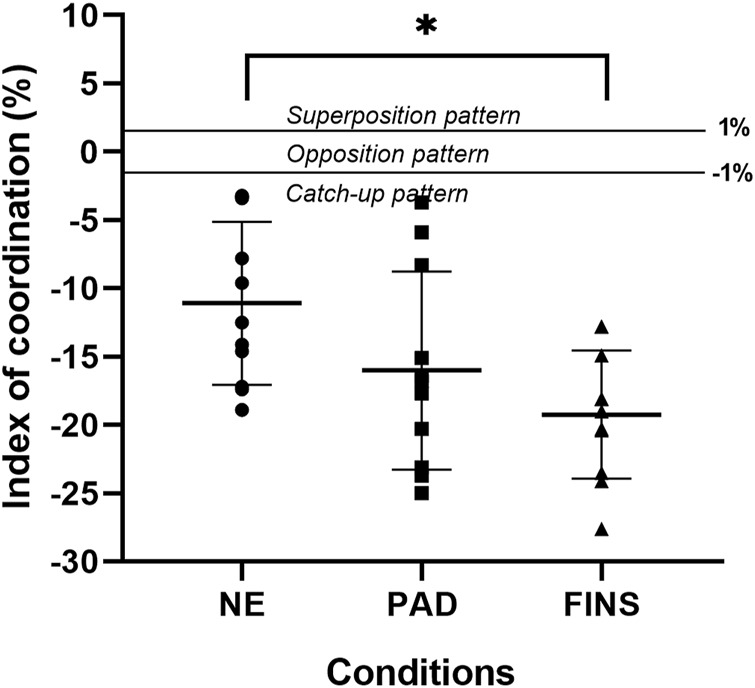
Mean ± SD of index of coordination (IdC, in % of total stroke cycle duration) in the no-equipment (NE), paddles (PAD) and fins (FINS) conditions; F_2, 20_ = 8.51; *p* = 0.002; partial eta^2^ = 0.46. * = FINS significantly lower than NE at *p* < 0.05.

**FIGURE 7:**
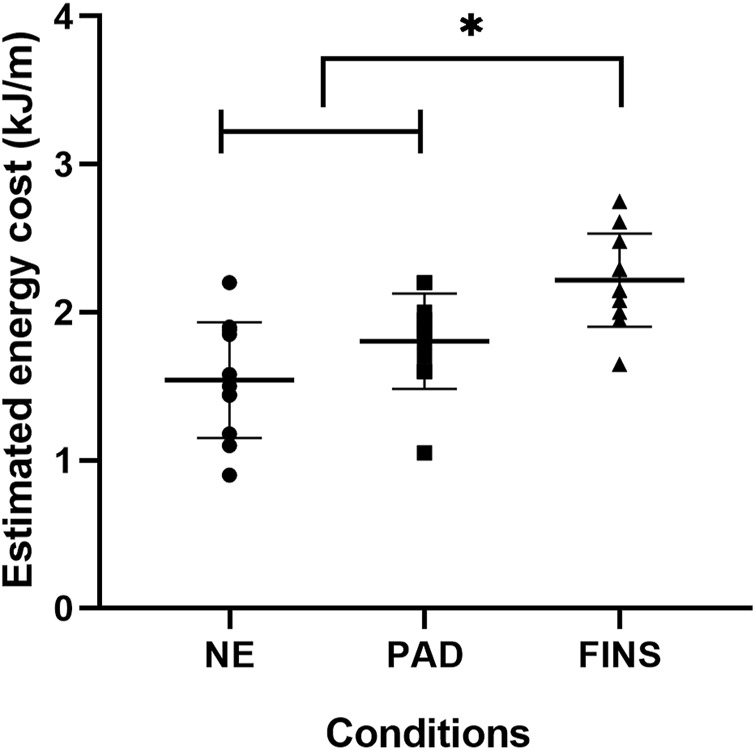
Mean ± SD of energy cost (C) in the no-equipment (NE), paddles (PAD) and fins (FINS) conditions; F_2, 20_ = 13.3; *p* < 0.001; partial eta^2^ = 0.57. * = FINS significantly higher than NE and PAD at *p* < 0.05.

Figures 4–6 present, respectively, the arm stroke efficiency ( ηp ), the propulsive time (Tprop), and the index of coordination (IdC) results. For the ηp (Figure 4), values measured for PAD or FINS conditions were significantly higher than those reported in NE (*p* < 0.05). The mean ± SD for Tprop during FINS was significantly lower (2.93 ± 0.23 s) than during NE (4.57 ± 0.24 s) (*p* < 0.05) and PAD (3.62 ± 0.28 s) (*p* < 0.05) (Figure 5). The IdC values were systematically in the catch-up pattern (Figure 6). Specifically, IdC values for NE were significantly higher (i.e., closest to the −1% limit associated to catch-up coordination pattern) than those computed for FINS (*p* < 0.05), but no difference was noted between PAD vs. NE and PAD vs. FINS. Finally, the C (Figure 7) was significantly higher during FINS (2.21 ± 0.31 kJ/m) than during NE (1.54 ± 0.39 kJ/m) (*p* < 0.05) and PAD (1.80 ± 0.32 kJ/m) (*p* < 0.05).

## 4 Discussion

Assuming paddles and fins as a task constraint in front crawl swimming, this study investigated the precise effects of each of this equipment on the kinematics, arm stroke efficiency, and coordination pattern parameters of the front crawl in 50 m all-out test. We first highlighted that the use of fins conducted to higher swimming velocity and therefore lower time to cover the imposed distance in comparison to PAD and NE trials. This may be explained by a larger SL and a lower kick amplitude than in PAD and NE trials. With FINS, swimmers exhibited a higher entry and catch duration, yet a lower propulsive time during one stroke, and these changes in the structure of the stroke cycle conducted to lower IdC values than during NE condition. Despite these coordinative changes, FINS and PAD demonstrated higher arm stroke efficiency than swimming without equipment. The higher swimming velocity with fins is also responsible for the higher C value found with FINS compared to NE and PAD.

The oral instruction given to the swimmers is to cover each 50 m at maximum speed, the time to cover the given distance is a first indicator of the achievement of the task. Regarding that variable with paddles, this presented similar results when compared to swimming without equipment whereas that time was lower in FINS. However, the adjustments adopted with equipment must be maintained for swimming without equipment to improve performance during training sessions. The use of equipment should be thought of as another tool to help the coach and the athlete to reach certain adjustments in the training sessions to obtain the improvement of the performance. Fins in the present study increased the foot area by 25%, representing a small increase in the propulsion area in the lower limbs compared to other studies that used this equipment, such as those by Zamparo et al. (2002); Zamparo et al. (2005); Zamparo et al. (2006) who observed increases from 108% to 309%. Despite being small fins (the present study), this caused changes in the time to cover the given distance and speed compared to swimming without equipment, as an acute effect. Fins increased SL, decreased KD, and AMP in comparison to other trials (NE and PAD). Fins with larger areas tend to significantly affect the mechanics of the lower limbs, decreasing KR (Pendergast et al., 1996; Zamparo et al., 2002; Samimy et al., 2005; Zamparo et al., 2005; Zamparo et al., 2006) as well as decreasing SR (Zamparo et al., 2005). Therefore, our study confirms what is empirically done in training, specifically the privileged use of small fins (*versus* larger ones) to get as close as possible to the situation of swimming without equipment, while allowing them to swim in training at competitive speeds. Higher percentage increases in the foot’s area with fins than those used in the present study could cause greater adaptations to athletes in relation to SL and SR and, consequently, in final propulsion. However, these increases could hinder the swimmers’ technique by causing unwanted adaptations, such as increases in the degree of flexion of the tibiofemoral joint, acetabulum-femoral joint. On the other hand, long-term training with fins with an area like the swimmer’s foot area can probably cause changes in the athlete’s v and improve body support, which can help reduce drag and change SL and SR (Zamparo et al., 2002; Zamparo et al., 2005; Zamparo et al., 2006).

Fins have also been used as a form of training to cause an increase in stroke frequency, seeking neural adaptations that allow an increase in final speed, in a form of assisted speed training, such as the use of extensors in swimming (Maglischo, 2003). However, this increase in v causes increases in drag, as active drag is proportional to speed squared (Kjendlie et al., 2004). Given the use of fins, swimmers should limit this increase in drag by acquiring a better horizontal body alignment provided by this equipment during the performance. Indeed, the more horizontal the body is, the better the hydrodynamic position of the body and the lower the drag (Marinho et al., 2009). Thus, as observed, the improvement in the time to cover the 50 m (the improvement in v) may be probably explained from a better body alignment causing less drag and greater propulsion from the lower limbs (Zamparo et al., 2002; Truijens and Toussaint, 2005). In other words, considering that the forward body displacement is obtained by subtracting drag to propulsive forces developed by the swimmer, the resulting swimming speed will be greater with fins.

Regarding the effects of the paddles, their surface area may have been the factor that determined the difference between PAD and NE conditions in SL and SR (i.e., higher SL and lower SR in PAD). Paddles increase the propulsive surface, so the swimmer can push a greater mass of water (Sidney et al., 2001). In the present study, the mean area of the swimmers’ hand was 149 cm^2^ and the area of the paddles was 300 cm^2^, which corresponded to an average increase of 101% of the hand’s area to generate propulsion. This increase was enough to impose many adjustments to athletes’ swimming mechanics compared to swimming without equipment. The results observed in the present study were like those that used paddles from 286 to 462 cm^2^ area, with hand area from 131 to 152 cm^2^ (Lerda and Chrétien, 1996; Gourgoulis et al., 2008; Gourgoulis et al., 2009; Telles et al., 2011).

Unlike the present study, Gourgoulis et al. (2008), Gourgoulis et al. (2009) and Lerda and Chrétien (1996) analyzed female swimmers. Although Seifert et al. (2004) mentioned that men and women have different adaptations in swimming mechanics (men develop higher SL both in maximum speed and 50-m freestyle event), these differences do not guarantee an improvement in performance, as it is related to the adopted technique. We can observe that regardless of gender, increases of 100% of the hand area are sensitive to alter the athlete’s swimming mechanics. Still, these alterations must be stimulated and preserved when the equipment is withdrawn. Paddles increase the propulsive force of the upper limbs, even at maximum intensity, and tend to significantly decrease the speed of the hand during the propulsive phases. They produce greater thrust (product between applied force and force application time), which combined with longer force application time, allows the swimmers to maintain or increase the swimming v at the expense of greater SL (Gourgoulis et al., 2008).

Paddles should be used with some caution so as not to cause adaptations that slow down the athletes’ movements, causing deleterious adaptations in performance. Both coaches and athletes should make progressive increments in paddle’s surface area so that athletes can adapt to this increase and transfer these changes from SL increases, SR decreases and potentially stroke phases durations modifications to unequipped swimming. Regarding this latter point from NE to PAD comparisons, the present study did not find any differences between the percentage duration of the stroke phases. In the literature, three studies (Gourgoulis et al., 2008; Gourgoulis et al., 2009; Telles et al., 2011), to the best of our knowledge, investigated the duration of the stroke phases according to the use of paddles. Similar results were found by Gourgoulis et al. (2008) and Telles et al. (2011). They reported that, although absolute increases were observed in the values of the duration of the phases, these were not reflected in the percentages of the relative duration. Gourgoulis et al. (2008) did not observe differences in the percentages of the stroke phases when comparing the swimming with paddles with the swimming without equipment. These authors report that the maintenance of the relative duration of the phases may be related to the size of the equipment used, larger paddles would cause changes in the duration of the propulsive and non-propulsive phases. Indeed, Gourgoulis et al. (2009) found increases in the percentage values of the duration of entry and catch with medium size paddles (286 cm^2^) when compared with small paddles (116 cm^2^), and swimming without equipment. However, Telles et al. (2011) used paddles of greater area than Gourgoulis et al. (2008) and found no changes in the duration of the propulsive and non-propulsive phases.

Considering the duration of the propulsive phases, two hypotheses can be formulated: (i) increment of applied force, without modification of the force application time (for example, with paddles, athletes could apply more force per stroke by displacing a greater volume of water backwards, so according to Newton’s third law - action and reaction—the swimmer will be displaced with more force forward); and (ii) increase in the time of force application (greater percentage duration of the propulsive phases of the stroke, the swimmer could apply force for a longer time) and different combinations of force and time. This study did not measure propulsive force, only variables that can be changed depending on the applied force, such as v, SL, and SR.

A factor that must be observed is the propulsion production of lower and upper limbs: the propulsion generated by upper limbs is greater (80%–90% of the total) when compared to the propulsion generated by the lower limbs (20%–10% of the total), in front crawl (Deschodt et al., 1999). Thus, equipment used (depending on the ratio of body area and percentage increase in the equipment) in lower limbs can provide improvements by reducing the body’s area of contact with the water, leaving the athlete in a more streamlined position. Furthermore, fins tend to change the mechanics of lower limbs, as observed in the present study. With fins, it increased the percentage time of the entry and catch phase (in comparison to both NE and PAD) and the recovery phase (in comparison to NE), that is, it significantly increased the percentage time of the non-propulsive phases. On the other hand, it decreased the percentage of the pull phase (in comparison to NE), causing a decrease in the percentage of the propulsive phases. Athletes with fins tend to benefit from greater body alignment and less drag by adjusting upper limb swimming mechanics, which was supported by lower SR to higher SL. The results of the present study were similar to those observed by Zamparo et al. (2005) where fins’ surface area ranged from 800 to 1,100 cm^2^ and the v evaluated were 1.0, 1.1, 1.2, 1.3 and 1.4 m/s. With fins, for front-crawl stroke, less energy is expended due to the reduction of the mechanical work of the upper limbs in locomotion in the water to cover a given distance (Zamparo et al., 2005).

Paddles and fins were not able, at maximum intensity, to change the coordinative pattern adopted by the swimmers in the present study (it remained in catch-up pattern). Only few of them presented values closed to −1% (i.e., opposition coordination pattern, according to Seifert, 2010) either in NE or PAD conditions. With fins, due to the lower continuity of the generation of propulsion (i.e., notably the larger entry and catch duration in comparison to NE and PAD, or the larger recovery duration in comparison to NE), there was a more pronounced catch-up pattern. As observed by partial eta^2^ = 0.46, the equipment had a great influence on this result (main IdC values were higher for NE than for FINS condition). To the best of our knowledge, only two articles compared the IdC of front crawl without equipment with swimming with paddles (Gourgoulis et al., 2009; Telles et al., 2011). Telles et al. (2011) analyzed front-crawl coordination pattern eliminating the effect of breathing. As a result, it was found that the IdC changed from swimming without equipment to swimming with paddles (from catch-up, with a value of −2.3% ± 5.0%, to opposition, with a value of −0.2% ± 3.8%), however this difference was not statistically significant. At high v, above 1.8 m/s, no change in the values of IdC was observed, which tends to remain in catch-up pattern. This was confirmed by Gourgoulis et al. (2009) who did not find differences in IdC values between situations without equipment (−10.03% ± 3.96%) with small paddles (−10.42% ± 4.15%) and with medium ones (−10.19% ± 4.21%). A catch-up coordination pattern is characterized by large intracyclic velocity variations due to the discontinuities in upper limb propulsive actions. However, Seifert et al. (2004) report that catch-up coordination pattern should not be characterized as poor pattern due to periods without propulsion, but simply the result of a different motor organization.

In addition, it should be considered that perhaps more important than the achieved swimming velocity, in relation to coordination patterns, would be the SR that allows reaching such velocity and would lead to changes in the IdC. Higher SR are usually reached by reducing the duration of the non-propulsive phases of the stroke (i.e., recovery and entry and catch), which would change IdC values closer to zero. It is noteworthy that the use of fins led to longer durations of the non-propulsive phases of the stroke compared to the NE condition.

The present study showed high ηp values with the use of fins and paddles compared to swimming without equipment. We can observe the high influence of the equipment by the high value of partial eta^2^ = 0.50. When we specifically analyzed the use of fins, the ηp value of the present study, 45%, was like that seen by Zamparo et al. (2005) ηp = 46%. In the current study, ηp was obtained using the equation by Zamparo et al. (2005) which uses data from v, SL and upper limb length, the present study also evaluated the maximal test with speeds that reached 2.10 ± 0.18 m/s. Pendergast et al. (2003) reported previous data from swimmers at submaximal speeds (1.0–1.4 m/s), and obtained the ηp through an equation fed with information from the MAD System. When compared to other activities such as running, the ηp in the aquatic environment has much lower values, however, the use of equipment tends to mitigate these differences. Coaches and athletes should make use of the adjustments promoted using this equipment to increase swimming performance without the use of these instruments. This would offer insights on the precise roles of such equipment to encourage its use at specific moments of training season, with well-defined functions.

With regard to energy cost, in front crawl stroke, it has an exponential relationship with swimming speed (Caputo et al., 2006). In the present study, the highest values of speed in the FINS condition led to the highest values of estimated energy cost. This needs to be considered when the swimmer uses FINS at supra-maximal swimming speeds to actually cause changes in the energy expended to move the body forward (Matos et al., 2013). In other words, the use of FINS at low swimming speeds would contribute little to the physiological adaptations resulting from the increased drag that occurs at high swimming speeds (Truijens and Toussaint, 2005).

Whereas in swimming paddles and fins are only used for training because are forbidden in competition, a recent sport activity such as “SwimRun” allows the use of this equipment. Indeed, SwimRunners repeat swimming and running courses, keeping their clothes (shoes and wetsuit) and materials. So, the results of this study could be used by the coaches and swimmers in training but also transferred to competitive events with higher stakes.

The present study has finally some limitations that should be mentioned. First, kinematics analyses were conducted at the center of the pool, on a volume of 10-m. This methodological restriction will be taken into consideration in a future study, where inertial sensors could be used, to enlarge the volume of analysis. Moreover, the position of the cameras used in the present study did not allow for obtaining a 3D view of the swimmers’ kinematics (specially to measure potential changes in the sculling movements performed with the equipment in comparison to NE). For this reason, the stroke phases cut was performed exclusively according to the work of Chollet et al. (2000), that defined front crawl stroke phases from a sagittal point of view. Even though the duration of the stroke phases and the IdC cannot be obtained without underwater images, it can be compensated by a simple use of a stopwatch leading to the acquisition of v, SR and SL. Therefore, these measurements should be encouraged between coaches and swimming teams, to verify the effects of the equipment on global kinematic parameters.

By comparing three all-out swimming conditions, the present paper found that the use of equipment such as fins deeply modify the structure of the front crawl stroke (from performance-related parameters through upper and lower limb kinematics to stroke efficiency and coordination pattern. The use of fins was associated with the lowest time to cover the given distance, inducing a higher energy cost compared to swimming with paddles, and swimming without equipment. Additionally, the most visible changes in the structure of the stroke cycle were noted in the FINS condition, with a longer duration of the non-propulsive phase (i.e., lower IdC values). From a practical point of view, coaches should therefore use equipment in a reasoned manner, varying its size (increasing or decreasing the force developed through the water), its frequency of use, and always staggering its use appropriately according to the objectives of the training session and the profile of the swimmer.

## Data Availability

The original contributions presented in the study are included in the article/Supplementary Material, further inquiries can be directed to the corresponding author.

## Abbreviations

AMP, kick vertical amplitude; C, energy cost; FINS, fins condition; IdC, Index of Coordination; KD, kick depth; KR, kick rate; NE, no-equipment; PAD, paddles condition; SL, stroke length; SR, stroke rate; Tcycle, mean cycle duration; Tprop, total propulsion time; v, mean swim velocity; ηp , arm stroke efficiency.

## References

[B1] Alberty M. Sidney M. Pelayo P. Toussaint H. M. (2009). Stroking characteristics during time to exhaustion tests. Med. Sci. Sports Exerc 41, 637–644. 10.1249/MSS.0b013e31818acfba 19204586

[B2] Amirsheybani H. R. Crecelius G. M. Timothy N. H. Pfeiffer M. Saggers G. C. Manders E. K. (2001). The natural history of the growth of the hand: I. Hand area as a percentage of body surface area. Plast. Reconstr. Surg. 107, 726–733. 10.1097/00006534-200103000-00012 11304598

[B3] Barbosa A. C. Castro F. D. S. Dopsaj M. Cunha S. A. Andries O. (2013). Acute responses of biomechanical parameters to different sizes of hand paddles in front-crawl stroke. J. Sports Sci. 31, 1015–1023. 10.1080/02640414.2012.762597 23360179

[B4] Caputo F. Oliveira M. F. M. Denadai B. S. Greco C. C. (2006). Intrinsic factors of the locomotion energy cost during swimming. Rev. Bras. Med. Esporte 12, 356–360.

[B5] Castro F. A. Correia R. Fiori J. M. Giuliano A. F. Trindade C. D. Z. Feitosa W. G. (2021). Practical application of the simplified model to assess the arm stroke efficiency: A tool for swimming coaches. Int. J. Perform. Anal. Sport 21, 900–908. 10.1080/24748668.2021.1957295

[B6] Chollet D. Chalies S. Chatard J. C. (2000). A new index of coordination for the crawl: Description and usefulness. Int. J. Sports Med. 21, 54–59. 10.1055/s-2000-8855 10683100

[B7] Cohen J. (1988). Statistical power analysis for the behavioral sciences. 2nd ed. Mahwah, NJ, USA: Lawrence Erlbaum Associates, Publishers.

[B8] Deschodt V. J. Arsac L. M. Rouard A. H. (1999). Relative contribution of arms and legs in humans to propulsion in 25-m sprint front-crawl swimming. Eur. J. Appl. Physiol. Occup. Physiol. 80, 192–199. 10.1007/s004210050581 10453920

[B9] di Prampero P. E. (1986). The energy cost of human locomotion on land and in water. Int. J. Sports Med. 7, 55–72. 10.1055/s-2008-1025736 3519480

[B10] Du Bois D. Du Bois E. F. (1916). A formula to estimate the approximate surface area if height and weight be known. Arch. Intern Med. 17, 863–871. 10.1001/archinte.1916.00080130010002 2520314

[B11] Faul F. Erdfelder E. Buchner A. Lang A.-G. (2009). Statistical power analyses using G*power 3.1: Tests for correlation and regression analyses. Behav. Res. Methods 41, 1149–1160. 10.3758/BRM.41.4.1149 19897823

[B12] Gourgoulis V. Aggeloussis N. Kasimatis P. Vezos N. Antoniou P. Mavromatis G. (2009). The influence of hand paddles on the arm coordination in female front crawl swimmers. J. Strength Cond. Res. 23, 735–740. 10.1519/JSC.0b013e3181a07357 19387407

[B13] Gourgoulis V. Aggeloussis N. Vezos N. Antoniou P. Mavromatis G. (2008). Hand orientation in hand paddle swimming. Int. J. Sports Med. 29, 429–434. 10.1055/s-2007-965570 17879890

[B14] Gourgoulis V. Aggeloussis N. Vezos N. Mavromatis G. (2006). Effect of two different sized hand paddles on the front crawl stroke kinematics. J. Sports Med. Phys. Fit. 46, 232–237. 16823353

[B15] Kjendlie P.-L. Stallman R. K. Stray-Gundersen J. (2004). Adults have lower stroke rate during submaximal front crawl swimming than children. Eur. J. Appl. Physiol. 91, 649–655. 10.1007/s00421-003-1021-1 14685866

[B16] Lerda R. Chrétien V. (1996). Speed-related changes in the spatiotemporal and physiological parameters of front crawl swimming with and without hand paddles. J. Hum. Mov. Stud. 31, 143–159.

[B17] Maglischo E. W. (2003). Swimming fastest. Champaign, IL, USA: Human kinetics.

[B18] Marinho D. A. Reis V. M. Alves F. B. Vilas-Boas J. P. Machado L. Silva A. J. (2009). Hydrodynamic drag during gliding in swimming. J. Appl. Biomech. 25, 253–257. 10.1123/jab.25.3.253 19827475

[B19] Matos C. C. Carvalho Barbosa A. De Souza Castro F. A. (2013). Utilização de palmares e nadadeiras no nado crawl: Respostas biomecânicas e fisiológicas. Rev. Bras. Cineantropom. Desempenho Hum. 15, 382–392. 10.5007/1980-0037.2013v15n3p382

[B20] Pendergast D. R. Tedesco M. Nawrocki D. M. Fisher N. M. (1996). Energetics of underwater swimming with SCUBA. Med. Sci. Sports Exerc 28, 573–580. 10.1097/00005768-199605000-00006 9148086

[B21] Pendergast D. Zamparo P. di Prampero P. E. Capelli C. Cerretelli P. Termin A. (2003). Energy balance of human locomotion in water. Eur. J. Appl. Physiol. 90, 377–386. 10.1007/s00421-003-0919-y 12955519

[B22] Ribeiro L. F. P. Lima M. C. S. Gobatto C. A. (2010). Changes in physiological and stroking parameters during interval swims at the slope of the d-t relationship. J. Sci. Med. Sport 13, 141–145. 10.1016/j.jsams.2008.10.001 19119067

[B23] Samimy S. Mollendorf J. C. Pendergast D. R. (2005). A theoretical and experimental analysis of diver technique in underwater fin swimming. Sports Eng. 8, 27–38. 10.1007/bf02844129

[B24] Sawyer S. F. (2009). Analysis of variance: The fundamental concepts. J. Man. Manip. Ther. 17, 27E–38E. 10.1179/jmt.2009.17.2.27E

[B25] Schnitzler C. Brazier T. Button C. Seifert L. Chollet D. (2011). Effect of velocity and added resistance on selected coordination and force parameters in front crawl. J. Strength Cond. Res. 25, 2681–2690. 10.1519/JSC.0b013e318207ef5e 21912344

[B26] Seifert L. Boulesteix L. Chollet D. (2004). Effect of gender on the adaptation of arm coordination in front crawl. Int. J. Sports Med. 25, 217–223. 10.1055/s-2003-45253 15088247

[B27] Seifert L. (2010). “Inter-limb coordination in swimming,” in *Biomechanics and Medicine in swimming XI*, 35–39. Editors Kjendlie P.-L. Stallman R. K. Cabri J. (Oslo, Norway: Norwegian School of Sport Sciences).

[B28] Sidney M. Paillette S. Hespel J. Chollet D. Pelayo P. (2001). “Effect of swim paddles on the intra-cyclic velocity variations and on the arm coordination of front crawl stroke,” in Proceedings of swim sessions of XIX symposium international on biomechanics in sports. Editors Blackwell J. R. Sanders R. H. (San Francisco, CA USA: Human Kinetics), 39–42.

[B29] Telles T. Barbosa A. C. Campos M. H. Junior O. A. (2011). Effect of hand paddles and parachute on the index of coordination of competitive crawl-strokers. J. Sports Sci. 29, 431–438. 10.1080/02640414.2010.523086 21259157

[B30] Truijens M. Toussaint H. (2005). Biomechanical aspects of peak performance in human swimming. Anim. Biol. 55, 17–40. 10.1163/1570756053276907

[B31] Yanai T. (2001). Rotational effect of buoyancy in frontcrawl: Does it really cause the legs to sink? J. Biomech. 34, 235–243. 10.1016/s0021-9290(00)00186-x 11165288

[B32] Yu C.-Y. Tu H.-H. (2009). Foot surface area database and estimation formula. Appl. Ergon. 40, 767–774. 10.1016/j.apergo.2008.08.004 18937935

[B33] Zamparo P. Cortesi M. Gatta G. (2020). The energy cost of swimming and its determinants. Eur. J. Appl. Physiol. 120, 41–66. 10.1007/s00421-019-04270-y 31807901

[B34] Zamparo P. Pendergast D. R. Mollendorf J. Termin A. Minetti A. E. (2005). An energy balance of front crawl. Eur. J. Appl. Physiol. 94, 134–144. 10.1007/s00421-004-1281-4 15702343

[B35] Zamparo P. Pendergast D. R. Termin A. Minetti A. E. (2006). Economy and efficiency of swimming at the surface with fins of different size and stiffness. Eur. J. Appl. Physiol. 96, 459–470. 10.1007/s00421-005-0075-7 16341874

[B36] Zamparo P. Pendergast D. R. Termin B. Minetti A. E. (2002). How fins affect the economy and efficiency of human swimming. J. Exp. Biol. 205, 2665–2676. 10.1242/jeb.205.17.2665 12151372

